# The Impact of Breastfeeding and Non-Nutritive Sucking Behaviors on Skeletal and Dental Malocclusions of Pediatric Patients: A Narrative Review of the Literature

**DOI:** 10.7759/cureus.19160

**Published:** 2021-10-31

**Authors:** Ahmed Almahrul, Lujain Alsulaimani, Faisal Alghamdi

**Affiliations:** 1 Advanced General Dentistry, The University Dental Hospital, King Abdulaziz University, Jeddah, SAU; 2 General Dentistry, Dental College, Ibn Sina National College for Medical Studies, Jeddah, SAU; 3 Oral Biology, Faculty of Dentistry, King Abdulaziz University, Jeddah, SAU

**Keywords:** review, orthodontics, dentition, bottle feeding, breast feeding, pediatric patients, sucking behavior, malocclusion, orofacial development

## Abstract

Studies suggest that breastfeeding can help resolve skeletal and dental malocclusions. But there is no clear evidence that breastfeeding duration affects skeletal or dental malocclusions in pediatric patients. Therefore, the objective of this narrative review was to review all the available updated articles on the impact of breastfeeding and non-nutritive sucking behaviors in deciduous and mixed teeth on different forms of malocclusions. The following electronic databases were used PubMed, Scopus, Web of Science, and Google Scholar to obtain relevant data that met the eligibility criteria. The studies in this narrative review were covered the last 10 years. Primary research identified 368 papers. In addition, three articles were selected from the gray literature for consideration. On the basis of duplication, title, abstract, methodology and/or irrelevant information, 177 articles were removed. This narrative review was focused on seventeen articles that met the eligibility criteria. When it comes to deciduous and mixed teeth, breastfeeding appears to decrease the occurrence of skeletal class II, posterior crossbite, and malocclusion. There appears to be a favorable correlation between prolong duration of breastfeeding and risk reduction. To avoid bias in the results, more longitudinal study is warranted, with data collected prospectively on the duration of exclusive breastfeeding and non-nutritive sucking behaviors, using specific survey questions, and subsequent clinical investigation of the occlusal status at the deciduous, mixed, and permanent teeth stages.

## Introduction and background

Only 40% of infants below the age of 6 months are exclusively breastfed globally. As the greatest source of nutrition for pediatrics, World Health Organization (WHO) actively encourages breastfeeding [1]. Functional triggers for example breathing, swallowing, sucking, and mastication are included in craniofacial development [2]. Breastfeeding, in addition to providing protection against infection, has also been shown to promote normal craniofacial development due to the high muscular activity required, which promotes appropriate lip closure, activates mandibular function, and locations the tongue properly against the palate [3]. The motions of the pediatrics lips and tongue during breastfeeding indicate that the child receives milk by a "squeeze action," but bottle-fed children make a more negative movement to get the milk, resulting in less activation of the orofacial structures [4]. Thus, breastfeeding can allow good occlusal development and proper orofacial structure growth, and the enhanced occlusal development can persist throughout the mixed teeth period. Nonetheless, the effect of breastfeeding on dental and skeletal occlusions remains debatable in the scientific literature.

Some researchers investigated the association between breastfeeding and malocclusion and arrived with very contradictory opinions, ranging from no association between breastfeeding and occlusion [5-7] to a specific relationship between a shorter period of breastfeeding and the presence of particular types of malocclusion, such as class II malocclusion [8,9], open bite [10], and cross-bite [11-14].

Because of this disagreement, the purpose of the current narrative review is to investigate the present evidence on the influence of breastfeeding and non-nutritive sucking behaviors on various dental and skeletal malocclusion problems of pediatric patients.

## Review

Methods

Literature Search Strategy

The search for this narrative review was done by using the following electronic databases: Scopus, Web of Science, Public Medline (PubMed), and Google Scholar digital data basis for publications in the English language was conducted from 2011 to 2021 owing to a lack of current and limited published reviews covering this area of research. In July 2021, a literature search strategy was established. Manual searching in the sources of included articles was done in order to find any studies that were missed during the initial search. The search was conducted using the following combination of keywords and Boolean operators ("AND", "OR"): [(breast) OR (feeding) OR (normal feeding) OR (breastfeeding) OR (breastfeeding)] AND [(sucking habit) OR (sucking behavior) OR (finger sucking) OR (thumb sucking)] AND [(infant) OR (infants) OR (newborn) OR (newborns) OR (neonates) OR (child) OR (children) OR (childhood) OR (pediatric) OR (pediatrics)] AND [(malocclusion) OR (occlusion) OR (skeletal malocclusion) OR (dental malocclusion) OR (angle classification ) OR (steiner's analysis)].

Eligibility Criteria

Only published papers that included a detailed description of the diagnostic method, information on non-nutritive sucking habits (such as thumb/finger sucking, tongue thrust, and pacifier use), newborn nutrition, breastfeeding types and duration, and diagnosis of malocclusion in the deciduous/mixed teeth of pediatric patients from zero to 15 years old were considered. Furthermore, the period time within the last 10 years (2011-2021) was considered in the search strategy. The following study designs were involved: case-control studies, randomized controlled trials (RCTs), and cohort studies. Articles that assessed the current evidence about the relationship between breastfeeding and pediatric malocclusion were included. Articles in non-human sources other than human sources, editorial or personal opinion articles, reviews, and articles with inadequate information on how the data was gathered were all excluded.

Data Extraction

The three reviewers were independently read the full articles and considered the following variables: title, abstract, methods, and main results. The data were then verified for completeness and accuracy and were harvested into a standardized Microsoft Office Excel worksheet. Data were gathered and organized into columns with the following information: study (author and year), sample size, data of breastfeeding types and their relationship with malocclusion, malocclusion diagnosis, time and instrument interval of feeding habit assessment, and treatment outcomes.

Study Selection

As a result of the keyword terms in the databases, a total of 368 articles were obtained. As a result of duplication or irrelevant subjects, 177 articles were removed, while 78 articles were removed based on the abstract and title of the article. Only 113 full-text articles were thoroughly screened for eligibility before these were included in the collection. Ninety-six of those papers were removed from our extensive analysis because they're either reviews (n=13), editorial or personal opinion articles (n=6), articles with inadequate information about how the data was gathered (n=47), or non-human sources (n=30). Lastly, 17 articles were included in this review. For this review, researchers used a literature search strategy outlined in Table 1.

**Table 1:** Summary of literature search strategy.

Search strategy		No. of publications
1	Publications retrieved from PubMed database	97
2	Publications retrieved from Scopus database	79
3	Publications retrieved from Web of Science database	55
4	Publications retrieved from Google Scholar	134
5	Publications retrieved through manual search	3
6	Total number of publications from electronic search (1+2+3+4+5)	368
7	Total number of publications after removal of duplicates	177
8	Publications remaining after title and/or abstract screening	113
9	Total included publications	17

Results

Study Characteristics

This review included 17 studies according to the eligibility criteria [6-8,11,12,14-25]. These studies were twelve cross-sectional studies, three were cohort studies, one was prospective study, and one was observational study. The total study sample included 11,054 pediatric patients ranging in sample size and age from 80 to 2060 human subjects and from 30th months to 15 years old, respectively with an average age between three and five years. The studies were conducted in different countries of the world; Australia, Brazil, China, France, India, Poland, and Spain. The other study characteristics (including malocclusion types considered, orthodontic diagnosis, assessment method, and samples’ age at the diagnosis of malocclusion) were collected and outlined in Table 2.

**Table 2:** Details of the included studies. CSS: cross-sectional study, OS: observational study, CS: cohort study, PS: prospective study, DS: doesn’t specify, NR: not reported, Y: years, M: months, CB: crossbite, OB: open bite, PCB: posterior crossbite, ACB: anterior crossbite, AOB: anterior open bite, ADB: anterior deep bite, OB*: overbite, OJ: overjet, IMW: intermolar width, ICW: intercanine width, IMD: intermolar distance, ICD: intercanine distance, WHO: World Health Organization.

Author/Country/ (Study Design)	Year	Sample Size	Samples’ Age at the Diagnosis of Malocclusion	Orthodontic Diagnosis	Malocclusion Types Considered	Assessment Method
Raftowicz-Wójcik et al. [6], Poland (CSS)	2011	245	3-5 Y	Clinical evaluations.	OB, OB*, and mesial bite.	Validated survey questions.
Lopes-Freire et al. [7], France, (OS)	2015	275	3-6 Y	Clinical investigation. Visual inspection for vertical and transverse relation. Angle class, deciduous dentition canine relationship for inter-arch sagittal malocclusion.	OB, AOB, PCB, sagittal occlusal relationship (OJ, angle class, deciduous canine relationship).	Validated survey questions.
Caramez da Silva et al. [8], Brazil, (CSS)	2012	153	3-5 Y	The sagittal relation between the maxilla and mandible were assessed by direct clinical investigation. Malocclusions were examined according to Foster and Hamilton’s classification.	Deciduous dentition, sagittal relationship.	Trained research assistants conducted telephone or in-person interviews to collect data on nutritional and non-nutritive sucking behaviors at 7, 30, 60, 120, and 180 days.
Peres et al. [11], Australia, (CS)	2015	1303	5 Y	Clinical investigation in a single home visit. WHO classification.	OJ, CB, and OB.	Survey questions reported at 3, 12, and 24 M.
Chen et al. [12], China, (CSS)	2015	734	3-6 Y	DS	OB, ACB, PCB, deep bite, sagittal relationship between 2^nd^ deciduous molars and between deciduous canines, absence or presence of spacing or crowding.	For the first 3Y of life, the survey questions were performed every 6 M, as were the non- nutritive sucking behaviors.
Limeira et al. [14], Brazil, (CSS)	2014	714	6-9 Y	The clinical investigation was carried out with the participant sitting in a chair and under good lighting, with disposable tongue blades and gloves. In centric occlusion, the occlusal relationships were assessed.	PCB	Guardians answered a standardized survey on the period of time they were solely breast-fed and the period of breast-feeding.
Campos et al. [15], Brazil, (CS)	2018	290	6 Y	Clinical investigation. AOB was examined according to the criteria given by Foster and Hamilton classification.	Skeletal and dental malocclusions were assessed.	The Z-score development index (height/age ratio) was applied to indicate nutritional status at birth and at six years old (WHO reference standard).
Sum et al. [16], China, (CSS)	2015	851	2-5 Y	Established their own criteria.	Sagittal relationship; incisal relationship between 2^nd^ deciduous molars, and between deciduous canines, OB*, AOB, PCB, ACB, IMW, ICW, and OJ.	Self-administered survey questions on history of non-nutritive sucking behaviors and feeding behaviors.
Agarwal et al. [17], India, (CSS)	2014	415	4-6 Y	Self-defined criteria.	PCB, AOB, and maxillary and mandibular ICD and IMD.	Validated survey questions.
Agarwal et al. [18], India, (CSS)	2016	415	4-6 Y	Clinical investigation.	AOB, OB*, malocclusion of deciduous teeth, and convex facial profile.	Self-administration survey questions on non-nutritive sucking behaviors and breastfeeding time period.
Moimaz et al. [19], Brazil, (PS)	2014	80	30^th^ M of age	NR	OJ, PCB.	Self-administered surveys at 1Y, 1.5Y, and 2Y of age.
Romero et al. [20], Brazil, (CSS)	2011	1377	3-6 Y	Clinical investigations were done by visual inspection.	OB* alterations: AOB and ADB.	Validated survey questions.
Costa et al. [21], Brazil, (CSS)	2018	489	2-5 Y	WHO index	Median line deviation, OB, and spacing or crowding.	Validated survey questions.
Germa et al. [22], France, (CS)	2016	422	3Y	Clinical investigation. Direct checkup for vertical and transverse relation.	PCB, AOB.	Self-administered survey questions.
Thomaz et al. [23], Brazil, (CSS)	2012	2060	12-15 Y	Facial characteristics and malocclusions were assessed as defined by Angle classification.	All the three classes of Angle classification.	Validated survey questions.
Jabbar et al. [24], Brazil, (CSS)	2011	911	3-6 Y	Self-defined criteria.	Increased of ACB. Deciduous canine relationships.	Validated survey questions.
Boronat-Catalá et al. [25], Spain, (CSS)	2019	320	8-11 Y	Clinical investigations.	OB,AOB, PCB,OJ, OB*, midline displacement, spacing, diastema, crowding.	Validated questionnaire and index assessment

The Influence of Breastfeeding on Development Different Types of Skeletal and Dental Malocclusions

Regarding the transverse skeletal malocclusion, ten articles evaluated the association between maxillary hypoplasia (posterior crossbite) and bottle-feeding or breastfeeding as shown in Table 2. Some articles evaluated the aforementioned relationship in mixed teeth, focused their attention on the deciduous teeth [7,11,12,15-17,22]. Regarding the vertical discrepancy, eleven studies [6,7,11,12,15,16,18,20,21,22,23] examined the association between open bite or overbite and breastfeeding in primary dentition. On the other hand, regarding sagittal discrepancy, six articles [7,11,12,16,21,24] assessed the association between anterior crossbite and breastfeeding in primary teeth and mixed teeth [25].

One study [23] assessed the association between prevalence of class II and breastfeeding in mixed teeth, while another study [8] for the same association was done in deciduous teeth. In one of the included studies [14] discovered that the decreased or absence duration of breastfeeding could be a risk factor in the mixed teeth for posterior crossbite. On the other hand, regarding the primary teeth, some of the articles found an increased risk for lower bite force and posterior crossbite if the child was breastfed for less than 6 months or if it was not breastfed [11,12,17].

Regarding the conclusions on vertical discrepancy differ amongst the included studies in this review. One study [22] found no association between breastfeeding and anterior open bite, while two studies [11,20] found anterior open bite was related with a short duration of breastfeeding. One study [19] discovered that participants who had been breastfed for more than one year had a higher incidence of increased overbite. On the other hand, another study [16] found no link between vertical discrepancy and breastfeeding. In regards to sagittal disparity, few investigations have discovered a link between decreased overjet and prolonged breastfeeding [11,16]. However, one study [19] showed higher overjet in patients who had been breastfeeding for more than a year. Some investigations that assessed the effect of breastfeeding on dental class II showed that participants who had breastfed for a longer period of time were less prone to obtain this type of malocclusion in mixed teeth [23] and deciduous teeth [8].

In regards of breastfeeding and occlusion development, one study found that breastfeeding improves occlusion [11], while another study found that children who had not been breastfed were much more prone to developing malocclusion [15]. On the other hand, one study found no link between these two parameters [7]. According to another study [21], participants who were used a pacifier and never or not exclusively breastfed had greater malocclusion than those who were exclusively breastfed and didn’t have non-nutritive sucking behaviors. The authors hypothesized that the use of a pacifier would alter the relationship between breastfeeding and occlusal status.

Three studies [12,18,21] investigated the relationship between breastfeeding and the occurrence of diastemas in primary dentition. Two studies [12,18] discovered that breastfeeding for up to 6 months is linked with the lack of maxillary diastemas, but the third study discovered that breastfeeding is connected with primate spaces and diastema. On the other hand, no one study of the included studies in this review assessed the association between maxillofacial growth pattern and breastfeeding in mixed teeth.

Discussion

Breastfeeding as a Preventive Factor for Malocclusion Development

Breastfeeding has been linked to the development of dental arches in the temporal teeth in both the anterior sagittal and transverse planes [16,25]. Breastfeeding may have a preventative impact on the improvement of malocclusions because it supports appropriate growth, muscle and bone development [26]. Breastfeeding supports the newborn's normal nasal breathing during and after breast milk sucking, preventing oral breathing and therefore reducing the improvement of malocclusions [23,26]. As shown in the study by Sanchez et al. [27], who used Steiner and McNamara's and Ricketts cephalometric values to compare lateral skull radiographs of 197 participants (106 breastfed and 91 bottle-fed), they found that breastfeeding resulted in an improved sagittal and vertical sagittal relationship within the cranial base and maxilla in agreement line with outcomes of the other study [28].

Boronat-Catalá et al. [25] studied 320 Spanish participants assessed the relationship between the breastfeeding and occlusion. Therefore, it was discovered that the participants which obtained natural feeding for less than four months, had the largest percentage of participants with posterior crossbite. 851 Asian pediatric patients aged two to five years participated in a cross-sectional research done by Sum et al. in Hong Kong [16].

For the purpose of gathering information on non-nutritive sucking behaviors and breastfeeding, the parents of the participants in the experiment answered a questionnaire. Those who were exclusively breastfed for more than six months were less prone to developing a malocclusion in deciduous teeth, as they concluded that exclusive breastfeeding for more than six months is significantly linked with the eugnathic growth of the maxillomandibular complex in both the sagittal and transversal dimensions, meaning that participants are less prone to developing crossbite and maxillary hypoplasia [16]. Gomes et al. [28] conducted an experiment in Brazil to measure and compare the activity of the buccal, masseter, and temporal muscles during different types of baby feeding. To ease examines these variables, they divided a sample of 60 participants aged two to three months into three groups: (1) breastfeeding exclusively, (2) artificial feeding through bottle, and (3) artificial feeding by cup. During feeding, all of the participants received surface electromyography. The group that was exclusively breastfed was much more active in terms of rate of movement and average curtailment of the temporal and masseter muscles than the group that was solely bottle-fed. As for bucinatores, the only significant difference was the curtailment range, which was larger in the group that breastfed than in the group that consumed bottled feed.

The previous findings indicate that the temporary, buccinators, and masseter muscles of participants who were exclusively breastfed and those who were cup-fed had similar muscular activity; thus, cup feeding may be used as an alternative infant feeding technique since the hyperactivity of the buccal muscles enhances the bottle's action and might enhance the stomatognath and changes in structural growth [29, 30].

Breastfeeding Duration and Development of Parafunctional Habits

At least six months of breastfeeding, a systematic review published in 2017 concluded that breastfeeding prevents the development of unhealthy oral behaviors because of the psychological stability that comes from that suction sensation [31].

It was reported by Thomaz et al. in 2012 with a sample of 2060 participants aged 12-15, whose findings revealed a short period of breastfeeding (less than six months) with the development of malocclusions, indicating dental class II and oral breathing. Pediatric patients who have been breastfed for fewer than six months may be at risk of developing malocclusions as a result of a synergistic effect of breastfeeding and parafunctional behavior [23].

For example, Lopes et al. looked investigated the impacts of exclusive and mixed breastfeeding on non-nutritive sucking behaviors in 275 participants aged three to six years old. Of those, 28 had exclusively breastfed and 247 had mixed breastfed. 224 participants were found to have parafunctional behaviors (81.5%). In one study, they concluded by participants who exclusively breastfed won't develop parafunctional behaviors [7]. During the year 2007, Leite et al. performed a research with the aim of evaluating the association between malocclusion problems and the type of breastfeeding obtained with improvement of nonnutritive sucking behaviors [29]. The study included 342 participants between the ages of three and five years old and discovered that non-nutritive sucking behaviors were prevalent in 70%-77.4% of the group investigated. Malocclusions were found in 87% of the participants [29].

An 84.2% reported having had breastfeeding, and 79.9% of these participants had some indication of malocclusion during the clinical evaluation. There is a strong correlation between the duration of breastfeeding, the use of artificial milk, and the improvement of nonnutritive sucking behaviors in pediatric patients regarding the improvement of malocclusions. A 66% (n = 46) of the 70 participants who were breastfed for at least 19 months showed no unfavorable oral habits [29]. A study was conducted in Caracas with the aim of assessing the correlation between the development of parafunctional behaviors and breastfeeding for only a few short months by reviewing 195 medical records of participants aged between three and sixteen years old [30].

The form of breastfeeding obtained, the duration of breastfeeding, the existence of unfavorable oral behaviors (such as lingual interposition, digital sucking, atypical swallowing, pacifier usage, and bruxism), and the existence of malocclusions were all studied [32,33]. The scientists found a correlation between the development of parafunctional behaviors and breastfeeding time period of less than six months, with the risk being higher for participants who did not get breastfeeding [31].

Promoter of Malocclusions and Bottle-Feeding as an Etiological Factor and Harmful Oral behaviors

The scientific literature has shown that bottle feeding as an alternative to breastfeeding increases the risk of malocclusion. It was reported by Mendoza et al. in 2008 [34] that 500 Bolivian pediatric patients aged from 3 to 7 years participated in their study. Malocclusions can be prevented by breastfeeding in 1st of 6 months of the life, according to the investigators. Artificial feeding, on the other hand, is considered a risk factor for their development. A 64% of bottle-fed youngsters were found to engage in non-nutritive sucking behaviors, pacifier suction is the most common, followed by digital suction (53%), and other behaviors such as lingual interposition (28%) and lipstick (19%). According to the findings of the study, artificial feeding with non-nutritive sucking behaviors are the primary risk factors for malocclusions [34].

Using 100 children below the age of one year, Moimaz et al. (2008) evaluated the association between the development of non-nutritional sucking behaviors and infants feeding techniques [19]. Most of the pediatrics were being fed by breastfeeding, with 75% of the participants being breastfed. Digital sucking and pacifier sucking behaviors were observed in 55% of the participants, and 74% of the participants who were bottle-fed. A possible explanation for these findings is that participants non-nutritive sucking behaviors could be associated to artificial feeding.

It was observed by Chen et al. (2015) that artificial breastfeeding time impacts the development of malocclusions in 734 pediatric patients in Beijing [12]. The risk of maxillary compression, posterior crossbite, and canine Class II in pediatric patients who were bottle-fed for more than 18 months is greater than 1.6%, 1.16%, and 1.43%, respectively.

Due to the rigid material used in artificial lactation, which has a minimum functional requirement at the moment of feeding, artificial lactation can result in an insufficient mandibular development, resulting in a lack of muscular activation patterns, leading to transverse improvement of the palate and improper dental alignment, these conditions in which the prevalence of dental and skeletal malocclusions is strongly correlated.

## Conclusions

It can be concluded that this review is relevant in a present-day scenario with an increasing number of bottle-fed children. Dental occlusions and poorer facial muscular strength are often attributed to a lack of efficient and ample breastfeeding by infants. Breastfeeding duration at six months or longer decreases the incidence of posterior crossbite and class II malocclusion in deciduous and mixed teeth, according to the literature. However, the effectiveness of breastfeeding protection in reducing other kinds of malocclusion is unclear (e.g., vertical discrepancy like deep bite or open bite). To reduce biases and confusion caused factors by non-nutritive sucking behaviors, Longitudinal prospective researches with data on time period and other features of breastfeeding (e.g., mixed or exclusive breastfeeding, correlation with not nutritive sucking behaviors and so forth), followed by an assessment of the occlusal status during the deciduous, mixed, and permanent teeth would be extremely helpful.
